# 

**Published:** 1901-01-19

**Authors:** 


### NOTICE TO CORRESPONDENTS.

All MSS., Letters, Books for Review, and other matters intended for the Editor should be addressed The Editor, "The Hospital," The Hospital Building, 28 & 29 Southampton Street, Strand, W.C.

The Editor cannot undertake to return rejected MS., even when accompanied by stamped directed envelope.

**Notice to Advertisers and Subscribers.**

All Advertisements, Orders for Copies of The Hospital, and Business Communications should be addressed to **THE MANAGER (Not to the Editor),** The Hospital, 28 & 29 Southampton Street Strand, London, W.C.

The Cost of Subscription, post free, is as follows:—Per week, 2½d.; per quarter, 2s. 8d.; per half-year, 5s. 3d.; per annum, 10s. 6d. Foreign:—Per annum, 15s. 2d., payable, in all cases, in advance.

**NOTICE.—Vol. XXVIII. of "The Hospital" (April to September, 1900), in handsome cloth binding, is now on sale at the Office of this Journal, price, 7s. 6d. Cases for binding the Numbers contained in this Volume 1s. 6d. Index to Vol. XXVIII., 2½d., post free.**

**The Monthly Part for February will be ready on February 1st. Price 6d., by post 8d.**

### Scraps and Gleanings.

The street collections in Bournemouth on Hospital Saturday this year realised £85 odd.

The plans of Mr. Jas. Miller, of Glasgow, for the new Royal Infirmary, Glasgow, provide for a reconstruction scheme, at a cost of £241,000.

This year's Hospital Sunday at Aberdeen resulted in £1,112 being collected from 61 churches and meeting places, compared with £1,106 collected from 48 churches and meeting places last year.

The Princess of Wales has forwarded to the secretary of the British Home and Hospital for Incurables, at Streatham, the sum of £6 10s. 6d., being a further portion of part proceeds of the sale of Canon Fleming's sermon, "Recognition in Eternity," published by Messrs. Skeffington and Sons.

Hospital Sunday in Bristol has been fixed for January 27th. During the three years that the Sunday Fund has been in existence £4,019 has been given by the various congregations; 154 churches and chapels have promised to contribute this year.

A completely new system of heating is to be introduced into the Royal Hants City Hospital at a cost of £4,000. The new drainage system in the institution has been carried out at a cost of £4,000.

During the past year the receipts at the Victorian Convalescent Home, Bognor, have been £947 odd, and the expenditure has been £774 odd. The endowment fund has now been brought up to £11,000.

### The Student of Medicine.

### APPOINTMENTS.

**Ernest Bisset**, M.B., Ch.B.Aberd., appointed Assistant House Surgeon to the Royal Portsmouth Hospital. **Margaret M. Traill Christie**, M.D., B.S.Lond., D.P.H.Cantab., appointed Medical Superintendent of the Victoria Dufferin Hospital, Calcutta. **John Cunningham**, M.B., C.M., appointed Visiting Medical Superintendent to the Gergenti Inebriate Home, by the Glasgow Town Council. **Douglas E. Darbyshire**, M.B.Vict., M.R.C.S., L.R.C.P., appointed District Medical Officer, Carnarvon, Western Australia. **C. H. G. Gostwyck**, M.B., Ch.B.Edin., appointed Third Assistant Medical Officer to the Kent Lunatic Asylum, Chartham. **C. D. Ingle**, M.R.C.S., L.R.C.P.Lond., appointed District Officer of the Langport Union. **Arthur Webb Jones**, F.R.C.S.Eng., appointed House Surgeon to the Royal Portsmouth Hospital. **A. R. Nicholls**, M.R.C.S., L.R.C.P., appointed Medical of Health to the Langport Rural District. **W. M. Palmer**, M.R.C.S.Eng., L.R.C.P.Lond., L.S.A., appointed Medical Officer to Linton District and Workhouse. **S. Herbert Perry**, M.D.Lond., M.R.C.P.Lond., appointed Casualty Assistant Physician to the General Hospital, Birmingham. **John Phillips**, M.B.Edin., appointed Physician to the Royal Portsmouth Hospital. **F. P. Sarjant**, M.B., M.R.C.S., appointed District Medical Officer of the Chorlton Union. **S. A. Shiach**, M.D.Edin., appointed Medical Officer and Public Vaccinator for the St. Mellon's District of the Newport Union. **A. Thomson**, M.B., C.M.Edin., appointed Medical Officer for the Alveston District of the Stratford-on-Avon Union. **Philip Dymock Turner**, M.D.Lond., appointed Honorary Medical Officer to the Royal Isle of Wight Infirmary and County Hospital. **Arthur Whittome**, M.B., appointed House Surgeon to the North-Eastern Hospital for Children, Hackney Road, N.E.

